# Epigenetic Changes at the *Birc5* Promoter Induced by YM155 in Synovial Sarcoma

**DOI:** 10.3390/jcm8030408

**Published:** 2019-03-24

**Authors:** Aleksander Mika, Sarah E. Luelling, Adriene Pavek, Christopher Nartker, Alexandra L. Heyneman, Kevin B. Jones, Jared J. Barrott

**Affiliations:** 1 Departments of Orthopedics and Oncological Sciences, Huntsman Cancer Institute, University of Utah School of Medicine, Salt Lake City, UT 84112, USA; Aleksander_Mika@urmc.rochester.edu; 2 Department of Biomedical and Pharmaceutical Sciences, Idaho State University, Pocatello, ID 83209, USA; luelsara@isu.edu (S.E.L.); paveadri@isu.edu (A.P.); nartchri@isu.edu (C.N.); heynalex@isu.edu (A.L.H.)

**Keywords:** survivin, *Birc5*, synovial sarcoma, YM155, epigenetics

## Abstract

YM155 is an anti-cancer therapy that has advanced into 11 different human clinical trials to treat various cancers. This apoptosis-inducing therapy indirectly affects the protein levels of survivin (gene: *Birc5*), but the molecular underpinnings of the mechanism remain largely unknown. Synovial sarcoma is a rare soft-tissue malignancy with high protein expression of survivin. We investigated whether YM155 would be a viable therapeutic option to treat synovial sarcoma. YM155 therapy was applied to human synovial sarcoma cell lines and to a genetically engineered mouse model of synovial sarcoma. We discovered that YM155 exhibited nanomolar potency against human synovial sarcoma cell lines and the treated mice with synovial sarcoma demonstrated a 50% reduction in tumor volume compared to control treated mice. We further investigated the mechanism of action of YM155 by looking at the change of lysine modifications of the histone tails that were within 250 base pairs of the *Birc5* promoter. Using chromatin immunoprecipitation (ChIP)-qPCR, we discovered that the histone epigenetic marks of H3K27 for the *Birc5* promoter changed upon YM155 treatment. H3K27me3 and H3K27ac increased, but the net result was decreased *Birc5*/survivin expression. Furthermore, the combination of molecular events resulted in caspase 3/7/8 upregulation and death of the sarcoma cells.

## 1. Introduction

Synovial sarcoma is a soft-tissue malignancy with a predilection for adolescents and young adults. It is the fourth most common type of soft tissue sarcoma and accounts for 5–10% of all soft tissue sarcomas [1]. Synovial sarcoma is a well-established translocation-associated sarcoma and is defined by the presence of the t (X;18) (p11.2; q11.2) translocation, involving the *SS18* (formerly *SYT*) gene on chromosome 18 and one of several synovial sarcoma X (*SSX*) genes on chromosome X (usually *SSX1* or *SSX2*) [2]. Over 95% of synovial sarcomas can be characterized by expression of the *SS18-SSX* gene and it is used as a routine diagnostic marker for this type of cancer [3]. The protein product of the translocation-generated fusion gene interferes with assembly of BAF (BRG/BRM and associated factors) complexes, leading to widespread alterations in chromatin remodeling and cell state reprogramming [4]. The main driving genetic event in this cancer is known, yet no means to target the fusion protein *SS18-SSX* directly exists. Patients diagnosed with synovial sarcoma share the lack of therapeutic options of many other translocation-positive sarcomas.

Synovial sarcoma is generally considered a high-grade, aggressive sarcoma. However, progression can take several years as evidenced by an overall survival rate of 39% at 15 years [5]. The survival outcomes are much worse if the patient presents with metastatic disease at the time of diagnosis. At 5 years, the survival rate is 22% [5,6]. Targeted therapeutic strategies are both lacking and greatly needed. Currently, the available drug regimens to treat synovial sarcoma consist of traditional cytotoxic chemotherapies with relatively high response rates, but very limited ultimate effectiveness [7,8].

Survivin is a member of the inhibitor of apoptosis (IAP) gene family; its expression in tumors is associated with a more aggressive phenotype, shorter survival time, and decreased response to chemotherapy [9,10,11]. While it is clear that survivin restricts the execution of apoptosis, it has still not been fully resolved how survivin controls cell death [12]. In recent years, YM155 (sepantronium bromide) has been increasingly used as an anti-cancer agent due to its inhibitory effect on survivin. Despite the advancements of this drug in clinical trials, the mechanism whereby YM155 reduces survivin expression is also unknown. Reports differ on how YM155 might achieve indirect regulation of survivin, by epigenetically silencing *Birc5* at the promoter [13,14] or by altering posttranslational modifications that impact the stability of the survivin protein [15].

Our objective was to understand how YM155 induces synovial sarcoma cells to undergo apoptosis. In previous studies, YM155 has been found not only to affect the expression of survivin, but also the anti-apoptotic BCL2 family protein MCL1 in several tumor-derived cell lines [16]. These results have been reproduced for several other proteins including PI3-kinase, ERK, and STAT3 in PANC1 cells [17] and securin in glioblastoma [18]. Because YM155 is an effective pro-apoptotic compound, it has been difficult to determine if survivin expression is a direct effect of the drug on epigenetic regulation of the *Birc5* promoter or if it is a secondary consequence of activating other pro-apoptotic pathways in synovial sarcoma.

## 2. Experimental Section

### 2.1. Human and Mouse Expression Databases

All clinical trial data for YM155 were obtained through queries using the clinicaltrials.gov website on November 6, 2018. *BIRC5* gene expression in published cancer databases was established using the web-based data inquiry tool Metabolic gEne RApid Visualizer (MERAV) [19]. We restricted our search parameters to only those cancers represented in the YM155 human clinical trials. Mouse expression data were obtained by using the accession number GSE81476 from the NCBI GEO database.

### 2.2. Mice

Mouse experiments were conducted with the approval of the University of Utah’s Institutional Animal Care Committee in accordance with legal and ethical standards established by the National Research Council and published in the Guide for the Care and Use of Laboratory Animals (protocol # 14-01016). The previously described *Rosa26-LSL-SS18-SSX2* mice [20] were maintained on a mixed strain background, C57BL/6 and SvJ. Mice were genotyped with the following primers: *Rosa26-LSL-SS18-SSX2* (F flox-AAACCGCGAAGAGTTTGTCCTC, F wt–GTTATCAGTAAGGGAGCTGCAG-TGG, R-GGCGGATCACAAGCAATAATAACC). TATCre was dosed by 10 μL intramuscular injections at 50 μM at 1 month of age.

### 2.3. Cell Lines

The human SYO-1, Yamato, and HS-SY-II cell lines were received from Torsten Nielsen at the University of British Columbia. The MoJo cell line was developed as previously described from a consented patient’s synovial sarcoma [21]. Each was maintained in Dulbecco’s modified Eagle medium (DMEM) with 10% fetal bovine serum (FBS) and tested for mycoplasma using MycoAlert Plus (Lonza, Walkersville, MD, USA) every 6 months.

### 2.4. YM155 Mouse Treatments

Mice had tumors initiated at 1 month of age. Tumors developed for the next 6 months allowing tumor volumes to reach 500–1000 mm^3^. Mice were then subjected to daily intraperitoneal injections of 20 mg/kg of YM155 for 11 days. One mouse with bilateral tumors was censored at day 7. Tumors were measured using digital calipers and volumes were calculated using the equation (*L*∗*W*^2^)/2. A small portion of the tumor was fixed in 4% paraformaldehyde overnight and then embedded in paraffin. The rest of the tumor was flash frozen in liquid nitrogen and stored at −80 °C until further use.

### 2.5. MTT Viability Assay and caspase 3/7 and caspase 8 Enzymatic Activity

Cells were seeded in a 96-well tissue culture dish at 1 × 10^4^ cells per well. Twenty-four hours after seeding, serial dilutions of YM155 were applied so that the final concentration ranged from 10 μM to 1 nM. Between 24 to 48 h, MTT at 5 mg/mL was applied, and the cells were allowed to incubate at 37 °C and 5% CO_2_ for 2.5 h. Formazan crystals were resolubilized in 10% SDS and 0.01 M HCl. Absorbance was measured at 570 nm and normalized to 650 nm. Caspase activity was measured using the Caspase-Glo 3/7 and Caspase-Glo 8 (Promega, G8090, and G8200) assay systems. The manufacturer’s instructions were followed for a 96-well plate assay.

### 2.6. Immunohistochemistry and Immunoblotting

Paraffin-embedded tissues were stained by immunohistochemistry by rehydrating slides through a citrosolv and ethanol dilution wash. Antigen-retrieval was performed in 10 mM sodium citrate (pH 6.0). Sections were immunostained with the primary antibody anti-Survivin (Abcam, ab182132, 1:500) followed by horseradish peroxidase detection methods and counterstained with hematoxylin. Images were taken with a Leica DM6B Brightfield Microscope.

Protein was isolated from cells or tissues by incubating in a mild lysis buffer (10 mM Tris-HCl pH 8.1, 10 mM NaCl, 0.5% NP-40, and proteinase inhibitors). The fractions were clarified at 14,000 rpm for 10 min before loading 25–50 μg/sample for gel electrophoresis and transfer to PVDF membrane. Western blots were probed with the following primary antibodies: anti-Survivin (Cell Signaling Technology, 2808, 1:1000), anti-NFκB/p65 (Cell Signaling Technology, 8242, 1:800) anti-GAPDH (Proteintech, 10494-1-AP, 1:1000). The immunoblots were probed secondarily with goat anti-rabbit antibodies conjugated with horse radish peroxidase and bands were detected using Bio-Rad Gel Doc MP imaging station.

### 2.7. qPCR Birc5 Detection

Tumor DNA was harvested using DNeasy tissue and blood kit (Qiagen, 69504, Germantown, MD, USA). Quantitative PCR used the PerfeCTa SYBR Green FastMix (Quanta Biosciences, 95072-250, Gaithersburg, MD, USA) and a CFX Connect (Bio-Rad Laboratories, Hercules, CA, USA) for detection using the following primer sequences: mouse *Birc5* sense–ATCGCCACCTTCAAGAACTG and anti-sense–GGCCAAATCAGGCTCGTTCT; mouse *Gapdh* sense–TGTCAGCAATGCATCCTGCA and anti-sense–CCGTTCAGCTCTGGGATGAC; human *BIRC5* sense–GCCTGGCAGCCCTTTCTCAA and anti-sense–TCCCAGCCTTCCAGCTCCTT; human *GAPDH* sense–GTCAAGGCTGAGAACGGGAA and anti-sense–GCCTTCTCCATGGTGGTGAA.

### 2.8. Chromatin Immunoprecipitation–Quantitative PCR

Mouse tumors were pulverized in a Covaris tissueTUBE attached to a Covaris glass tube (13 × 65 mm) using a hammer and an aluminum block on dry ice. Samples were intermittently placed in liquid nitrogen to maintain molecular interactions. The powderized tissue was resuspended in PBS + protease inhibitors (Sigma, 11836153001, St. Louis, MO, USA) and fixed in 1% formaldehyde for 10 min at room temperature. The cross linking was quenched by adding 125 mM glycine. Samples were washed three times using cold PBS and cold centrifugation at 1000× *g* for 5 min. Similarly, cells can be fixed in 1% formaldehyde and washed while cells are still adherent to the bottom of the tissue culture dish. Cells in both cases were lysed using cold Farnham cell lysis buffer (5mM PIPES pH 8.0, 85 mM KCl, 0.5% NP-40) with protease inhibitors by incubating on ice for 10 min. Cells were collected and dounced with a glass tube and rod 20 times with a loose rod and 10 times with a tight rod. Samples were filtered through 70 μm filter and centrifuged at 1000× *g* for 10 min. The supernatant was removed and 1 mL of RIPA lysis buffer (1% NP-40, 0.5% Na deoxycholate, 0.1% SDS/PBS) was added for every 300 mg of tissue or 1 × 10^7^ cells and incubated on ice for 10 min. Sonication proceeded in the following manner: 1 mL aliquots were sonicated with a 30 s on and 30 s off cycle for 8 cycles totaling 4 min of sonication time. Sonicated samples were centrifuged at 1400 rpm for 5 min and the supernatant was kept for chromatin immunoprecipitation.

Dynabeads (Invitrogen, 11204D, Carlsbad, CA, USA) were washed in 0.5 mg/mL BSA/PBS three times on a magnet. Sonicated samples were precleared with 30 μL of beads for 1 h at 4 °C. After preclearing, 50 μL of the sample was set aside as the input DNA. Supernatants were transferred to a new tube and 5 μg of primary antibodies were added and incubated overnight at 4 °C. The following antibodies were used for immunoprecipitation. Anti-H3K27me3 (Rockland, 600-401-I84), anti-H3K27ac (Rockland, 600-401-K00), anti-H3K4me1 (Rockland, 600-401-I61), and anti-NFκB/p65 (Cell Signaling Technology, 8242). Samples were centrifuged at 10,000× *g* for 5 min and then combined with 100 μL of washed Dynabeads to be incubated for 4 h at 4 °C. Unbound sample was washed off six times with RIPA buffer, twice with LiCl buffer, and once with TE. 100 μL of elution buffer (1% SDS, 0.1 M NaHCO3, RNaseA) was added and incubated for 0.5 h at 37 °C followed by the addition of proteinase K and incubation at 37 °C for another 2.5 h. Samples were subsequently incubated at 65 °C for 9 h. DNA was purified using a DNA ChIP clean up and concentrator (Zymo, D5205, Irvine, CA, USA) following the manufacturer’s recommendations. Samples were diluted to 0.5 ng/μL and qPCR reactions were set up and run using the PerfeCTa SYBR Green FastMix and a CFX Connect for detection using the following primer sequences: human *BIRC5* promoter sense–GGGTGGATCACAAGGTCAGG and anti-sense–CACCACGCCTGGCTAATTTT; mouse *Birc5* promoter sense–CTGGCCAAATCCTGCAAACC and anti-sense–CTTTAAAGCATGTCCGCTGCA. The parameters for thermal cycling were 95 °C for 15 s for initiation followed by 40 cycles at 95 °C for 15 s followed by 60 °C for 60 s.

### 2.9. Statistical Methods

The control mice group with tumor was compared to the YM155 treated tumor group in a single factor ANOVA statistical test. For most comparisons between control and YM155 treated synovial sarcomas, Student’s t-tests were performed.

## 3. Results

### 3.1. Clinical Trials Data on YM155

The clincialtrials.gov website was searched to identify all previous clinical trials involving the use of YM155. Eleven registered phase 1 and 2 trials were identified. 405 patients with various forms of cancer were treated with YM155, with the earliest studies ending in 2007 and the most recent ending in 2015 (Table 1). Most of the clinical trials have not published or released their data. In the case of two clinical trials that were published the data supported not continuing the clinical trials due to serious adverse events or no statistical improvement over the standard of care [22,23]. While YM155 is associated with adverse events at doses above 4.8 mg/m^2^/day [24], proper combination therapies might be able to achieve efficacy and avoid toxicity-related events. 

### 3.2. Synovial Sarcoma Exhibits Similar BIRC5 Expression Levels to Other Cancers

**Figure 1:**
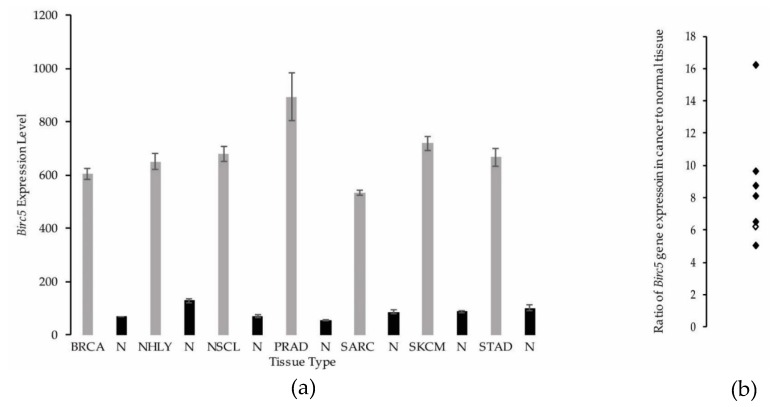
*BIRC5* gene expression in various cancer types. (**a**) Mean *BIRC5* gene expression in cancers (grey bars) and the corresponding mean in normal tissue (N; black bars). BRCA = breast invasive carcinoma (*n* = 190), NHLY = non-Hodgkin’s Lymphoma (*n* = 40), NSCL = non-small cell lung cancer (*n* = 65), PRAD = prostate adenocarcinoma (*n* = 15), SARC = synovial sarcoma (*n* = 3), SKCM = skin cutaneous melanoma (*n* = 156), STAD = stomach adenocarcinoma (*n* = 38); (**b**) Ratio of *BIRC5* gene expression in cancers to normal tissue. Synovial sarcoma is the open diamond. Means are represented ± SEM.

Using the web-based data inquiry tool Metabolic gEne RApid Visualizer (MERAV) [19], we searched human cancers that have been previously targeted in clinical trials using YM155 and compared the *BIRC5* gene expression levels in these cancers to synovial sarcoma (Figure 1a,b). We observed that synovial sarcoma was not dissimilar from other cancers in terms of their *BIRC5* gene expression nor in the ratio of gene expression found in the cancer compared to the corresponding normal tissue. This prompted us to pursue YM155 studies in synovial sarcoma.

### 3.3. Birc5/Survivin Expression in a Mouse Model of Synovial Sarcoma

**Figure 2:**
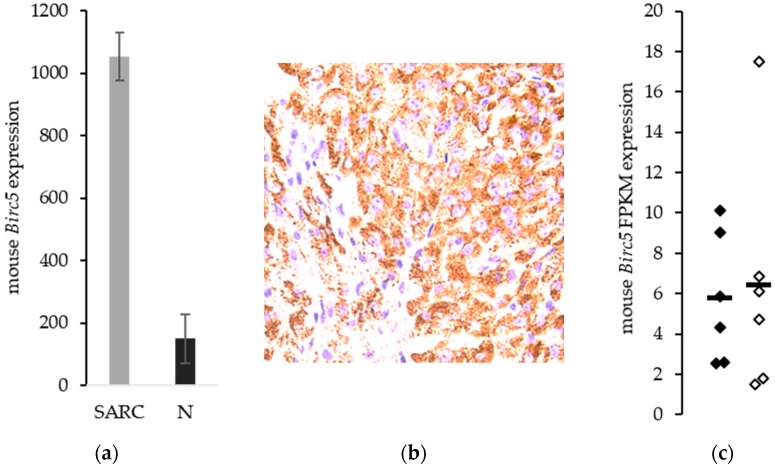
*Birc5*/Survivin expression in mouse models of synovial sarcoma. (**a**) Mean microarray expression for *Birc5* in mouse synovial sarcoma (*n* = 5) and mouse skeletal muscle (*n* = 4). (**b**) Survivin protein expression in murine synovial sarcoma. Scale bar = 50 μm. (**c**) *Birc5* gene expression in murine non-metastatic (◆) synovial sarcoma and metastatic (◊) synovial sarcoma. Bars represent means. Dashed red line represents normal skeletal muscle mean.

We have developed a genetically engineered mouse model of synovial sarcoma with a high correspondence to the pathophysiology of the human synovial sarcoma, including spontaneous generation of pulmonary metastases [20]. We evaluated our mouse models of synovial sarcoma for *Birc5* gene expression and found increased levels of transcript in the sarcoma tissue compared to normal skeletal muscle (*p* = 1.4 × 10^−4^). The ratio between *Birc5* expression in the mouse synovial sarcomas compared to the normal tissue was 7.0 and was very comparable to the 6-fold increase seen in the human comparison (Figure 2a). Immunohistochemical analysis of survivin demonstrated that the protein is expressed in these mice (Figure 2b). There was no difference in expression between the metastatic model and the non-metastatic model of synovial sarcoma. Mean FPKM (Fragments Per Kilobase of transcript per Million mapped reads) ± SEM for *Birc5* was 5.8 ± 1.3 and 6.4 ± 2.4, respectively (*p* = 0.82) (Figure 2c). These means in synovial sarcoma represent a log2 ratio of 5.3 or about a 40-fold increase in *Birc5* expression over mouse skeletal muscle. These values indicate that *Birc5* expression is not unique to the metastatic disease, but can be targeted equally in metastatic and non-metastatic synovial sarcomas.

#### 3.3.1. YM155 Inhibition of Human Synovial Sarcoma Cells in Vitro

**Figure 3:**
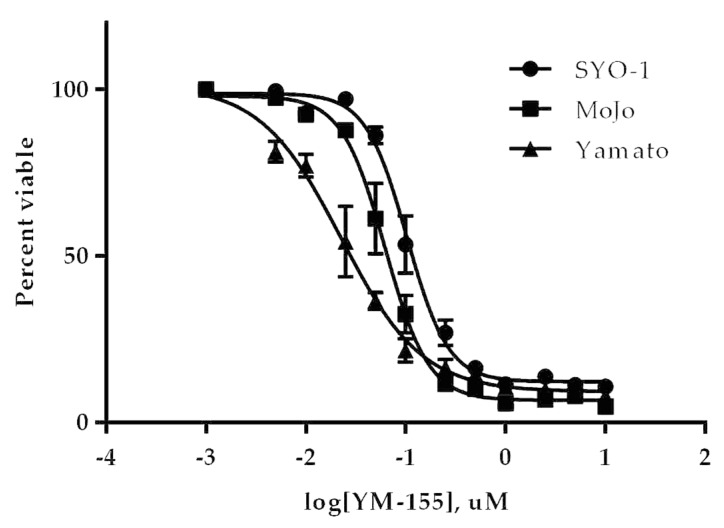
YM155 efficacy against human synovial sarcoma cell lines. Cells were treated for 48 h with various concentrations of YM155 and then assayed for the conversion of MTT to formazan and correlated to cell viability. YM155 showed the greatest potency in Yamato with an EC_50_ of 22 nM followed by Mojo and SYO-1 with EC_50_s of 62 nM and 103 nM, respectively.

**Figure A1:**
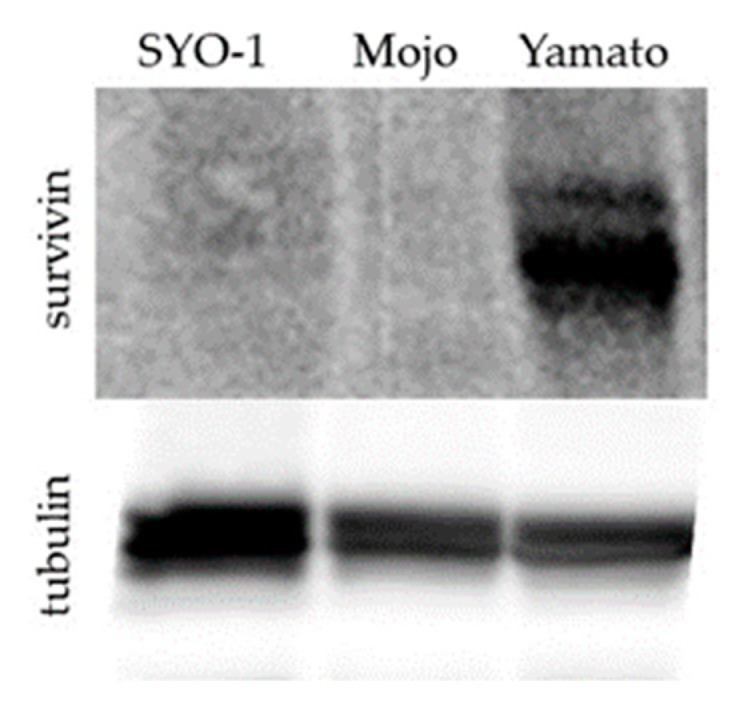
Immunoblot images of survivin and tubulin in human synovial sarcoma cell lines.

Three established human cell lines for synovial sarcoma were used to determine the EC_50_ of the inhibitor YM155 *in vitro*. After 48 h exposure to serial dilutions of YM155, similar nanomolar EC_50_ values were observed for all three cell lines (Figure 3). The Yamato cell line was the most responsive and also exhibited the most survivin protein level (Figure A1). This submicromolar potency prompted further investigation of YM155 in our mouse model of synovial sarcoma.

#### 3.3.2. YM155 Inhibition of Murine Synovial Sarcomas in Vivo

**Figure 4:**
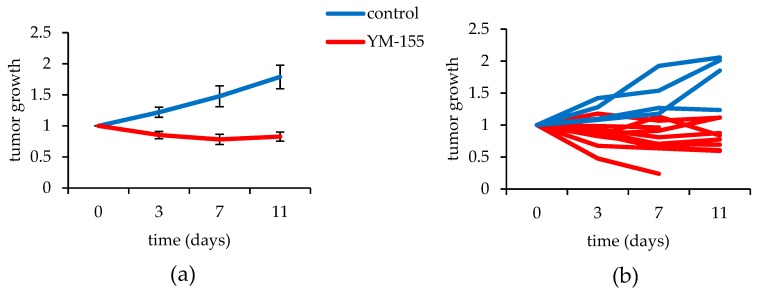
YM155 treatments of a mouse model of synovial sarcoma. (**a**) Mean values of tumor growth for mice treated with YM155 (red) and control treated (blue), bars represent the SEM; (**b**) Individual tumor growths represented for the 11 treated synovial sarcomas and the 4 control synovial sarcomas.

**Figure A2:**
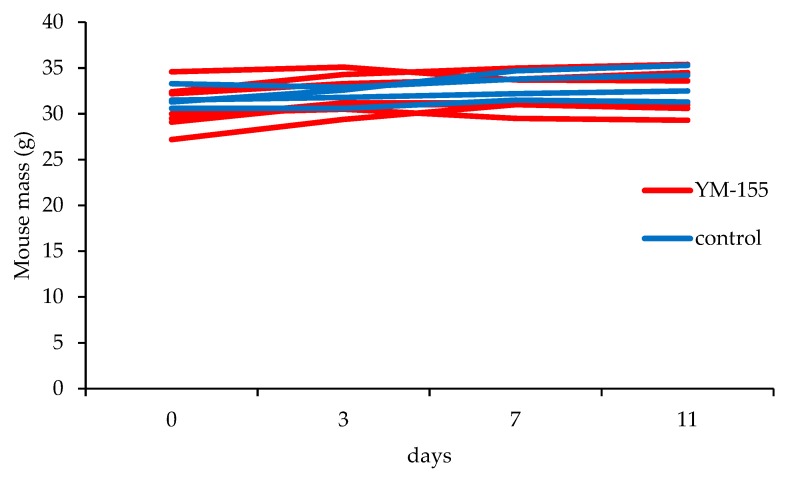
Weight changes in mice during the 11 days of YM155 treatment or control. No observable differences were seen for any individual mouse or between treatment groups.

Synovial sarcomas expressing the gene *Rosa26-LSL-SS18-SSX2* were induced with a localized injection of the protein TATCre in the anterior tibialis of the left hindlimb, right hindlimb, or both. Seven mice, accounting for 10 synovial sarcomas, were treated with 20 mg/kg of YM155 for 11 days. Four mice, accounting for 4 tumors, were treated with the vehicle control. The tumor growth was plotted against time and the mean growth rate of the control treated sarcomas exhibited a 1.78-fold increase over 11 days, whereas the YM155 treated tumors saw a 17% decrease during the same time period (Figure 4a,b). A single factor ANOVA revealed a statistically significant difference between the groups (*p* = 0.028). While one mouse was censored on day 7in the treatment group, there was no detectable weight loss in any of the YM155 treated mice (Figure A2), including the censored mouse that had such a dramatic response to the therapy before dying.

#### 3.3.3. The Effects of YM155 Inhibition in Murine Synovial Sarcoma on *Birc5*/Survivin

**Figure 5:**
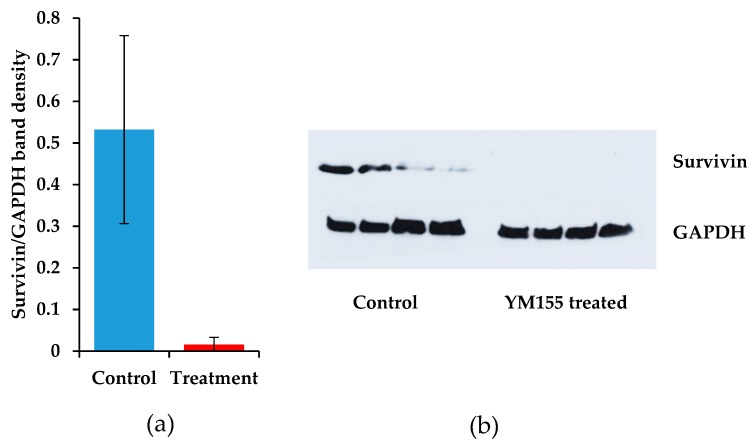
YM155 decreases survivin in mouse synovial sarcomas. (**a**) Survivin expression was immunoblotted and band density was measured. Survivin expression was normalized to GAPDH protein levels, bars represent the SEM, *n* = 4; (**b**) Immunoblots of survivin (top) and GAPDH (bottom) in control and YM155 treated mice after 11 days.

The most notable cellular response to YM155 therapy in most settings is characterized by decreased levels of survivin protein. We therefore measured survivin levels in our mice that were treated with YM155. Protein was extracted from sarcomas involved in the animal study of YM155. A striking decrease of survivin was measured after immunoblotting in the YM155 treated sarcomas. Despite the large variation in the control treated samples, the comparison between control and YM155 treated synovial sarcomas was significant (*p* = 0.034, Student’s *t*-test) (Figure 5a,b).

### 3.4. Establishing an in Vitro Model to Determine the Mechanism of Action of YM155

**Figure 6:**
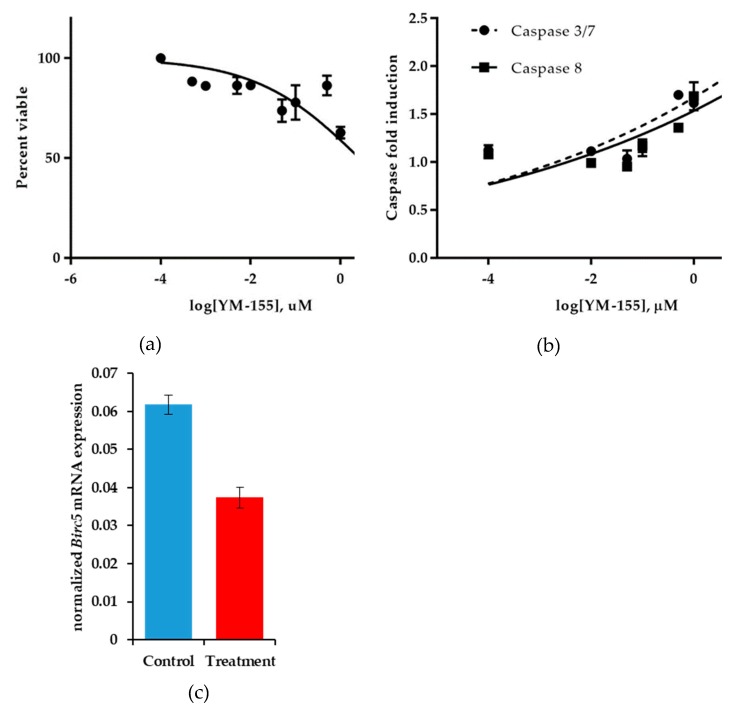
YM155 decreases viability and *BIRC5* expression in HS-SY-II cells. (**a**) Cells were treated for 24 h with various concentrations of YM155 and then assayed for the conversion of MTT to formazan and correlated to cell viability. (**b**) Caspase 3/7 & 8 activity detection in response to YM155 treatment. Fold increases are normalized to control treated HS-SY-II cells. Error bars are SDM. (**c**) Normalized mean values for *Birc5* expression. *BIRC5* was normalized with *GAPDH* mRNA expression, bars represent the SEM, *n* = 4 (*p* = 0.0006).

While it is noteworthy to discover another potential indication for the use of YM155 to treat cancers, we sought to understand better how YM155 exerts its negative effects on *Birc5*/Survivin expression. To determine the mechanism of action of YM155, we used another human synovial sarcoma cell line HS-SY-II. This cell line demonstrated a decreased sensitivity to YM155, albeit in a 24 h viability assay (EC_50_ = 2.64 ± 0.14 μM) compared to previously assayed cell lines over 48 h. However, during this shortened treatment duration, we observed decreases in *Birc5* expression corresponding increases in apoptosis (Figure 6a–c). Congruently, levels of caspase 3/7 and caspase 8 activity increased with higher concentrations of YM155 as detected by Caspase-Glo bioluminescent assays after 24 h incubation with YM155 (Figure 6b). Levels of *BIRC5* were measured by semi-quantitative PCR and transcript levels were normalized between control and YM155 treated samples using *GAPDH* mRNA expression. We observed a 39.5% reduction in normalized *BIRC5* expression in YM155 treated synovial sarcomas (*p* = 0.0006) (Figure 6c). HS-SY-II cells also proved to be the most effective synovial sarcoma cell line in terms of sample preparation and DNA fragmentation for chromatin immunoprecipitation assays.

### 3.5. Histone lysine Modifications Suggest an Epigenetic Mechanism of Action of YM155

**Figure 7:**
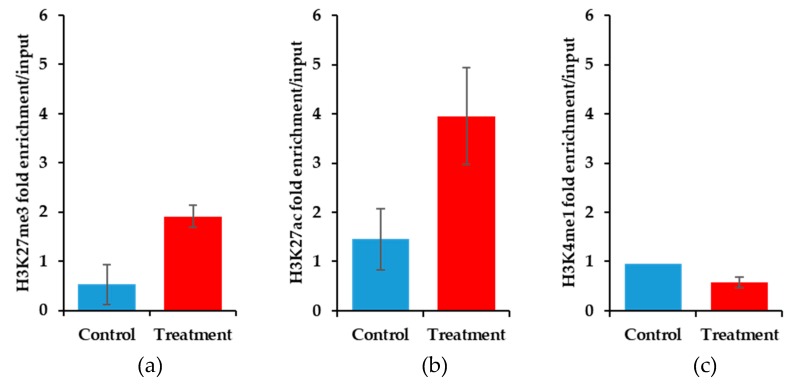
Epigenetic histone marks differ between control and YM155 treated mouse synovial sarcoma at the *Birc5* promoter. (**a**) ChIP-qPCR for H3K27me3 shows elevated levels at the *Birc5* promoter in the YM155 treated samples (*n* = 5) compared to controls (*n* = 3), error bars are SEM (*p* = 0.045). (**b**) ChIP-qPCR for H3K27ac shows elevated levels at the *Birc5* promoter in the YM155 treated samples (*n* = 5) compared to controls (*n* = 3), error bars are SEM (*p* = 0.14). (**c**) ChIP-qPCR for H3K4me1 shows equally low levels at the *Birc5* promoter between the YM155 treated samples (*n* = 5) compared to controls (*n* = 2), error bars for treated samples are SEM (*p* = 0.13).

**Figure A3:**
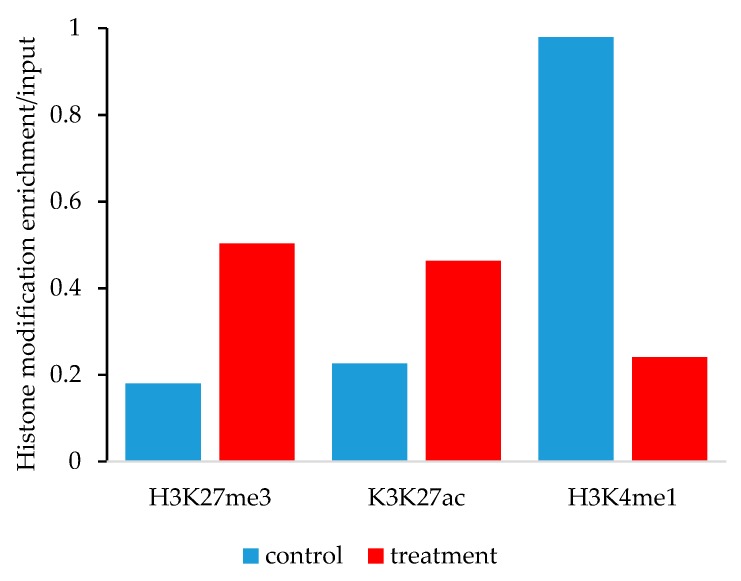
Epigenetic histone marks differ between control and YM155 treated human synovial sarcoma cells, HS-SY-II, at the *BIRC5* promoter. ChIP-qPCR for H3K27me3 shows elevated levels at the *BIRC5* promoter in the YM155 treated samples compared to controls. ChIP-qPCR for H3K27ac shows elevated levels at the *BIRC5* promoter in the YM155 treated samples compared to controls. ChIP-qPCR for H3K4me1 shows a decrease at the *BIRC5* promoter in the YM155 treated samples compared to controls.

Using the HS-SY-II human synovial sarcoma cell line, we sought to delineate the epigenetic mechanisms surrounding the *BIRC5* promoter in response to 0.2 μM YM155 after 24 h of treatment. The YM155 dose is 10-fold less than the EC_50_ measured for cell viability for 24 h so as to avoid globally induced artifacts of apoptosing cells. Cells were fractionated into nuclei with sonicated DNA in fragments that averaged 500 bp in size. DNA was immunoprecipitated using rabbit polyclonal antibodies, and PCR was performed using primers that were specific for the promoter region of the *BIRC5* gene. The treatment of HS-SY-II cells with YM155 resulted in an increase in enriched DNA in association with both H3K27 trimethylation and acetylation marks (Figure A3). We similarly performed PCR-chromatin immunoprecipitation on the mouse synovial sarcoma samples that were treated for 11 days with YM155. The concomitant enrichment for H3K27me3 and H3K27ac was also observed in these mouse synovial sarcomas (Figure 7a,b). Typically, we associate these signals as opposing one another, H3K27me3 leading to gene repression and H3K27ac leading to gene activation. We also studied the epigenetic mark, H3K4me1, and observed subtle decreases in the treated samples (Figure 7c). H3K4me1 is a modification that is context specific and can be associated with activated enhancer regions or synergistic repression when coupled with H3K27me3 [25,26,27]. While the mouse samples demonstrated a subtle decrease in H3K4me1 levels and the treated human cell lines exhibited a more dynamic decrease in enrichment (Figure A3). We next studied the levels of NFκB in response to YM155. NFκB is a putative transcription factor of the *BIRC5* gene [28].

### 3.6. The Transcription Factor NFκB Increases upon YM155 Treatment, but Fails to Activate Birc5

**Figure 8:**
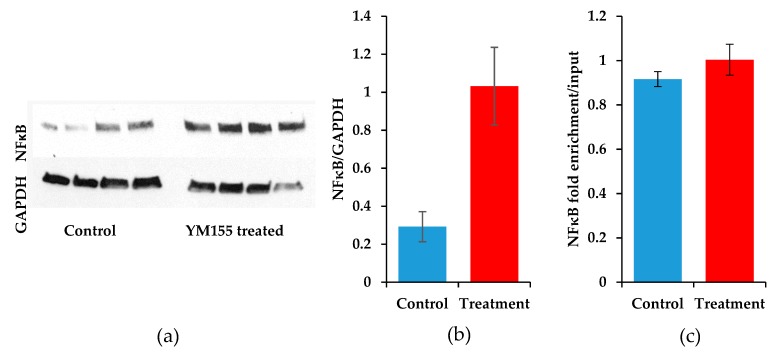
NFκB expression and *Birc5* promoter binding in response to YM155. (**a**) Immunoblots of NFκB expression (top) and GAPDH (bottom) in control and YM155 treated mice after 11 days. (**b**) Quantification of band intensities of NFκB normalized to GAPDH expression, error bars for samples are SEM (*p* = 0.015). (**c**) ChIP-qPCR for NFκB demonstrates near equal levels at the *Birc5* promoter between the YM155 treated HS-SY-II samples (*n* = 3) compared to controls (*n* = 3), error bars for treated samples are SEM (*p* = 0.32).

**Figure 9:**
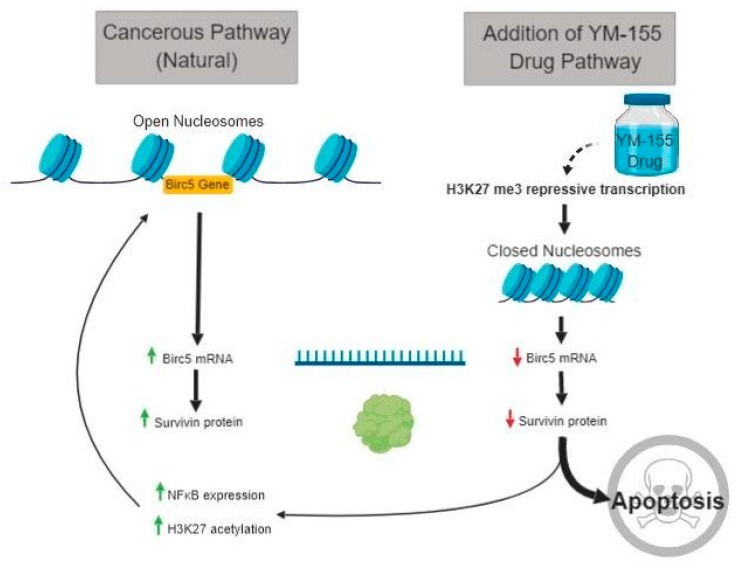
Survivin protein expression pathway in both control synovial sarcoma cells and YM155 treated synovial sarcoma cells.

To determine the level of input in the epigenetic histone modifcations of the transcription factor NFκB, we measured total protein levels and enrichment at the *BIRC5* promoter. Protein was extracted from sarcomas involved in the animal study of YM155. A striking increase of NFκB was measured after immunoblotting in the YM155 treated sarcomas (*p* = 0.015, Student’s *t*-test) (Figure 8a,b). Material to perform ChIP-qPCR on the mouse samples had been exhausted and thus we reverted back to the HS-SY-II cells treated for 24 h with 0.2 μM YM155. We were unable to detect a difference in the binding of NFκB to the *BIRC5* promoter in control and YM155 treated cells (Figure 8c). Therefore, despite the increase in survivin expression provided through a feedback loop mechanism, the repression of *BIRC5* as evidenced by H3K27me3 ChIP-qPCR and decreased survivin expression results in an apoptotic phenotype (Figure 9).

## 4. Discussion

Survivin is a viable clinical target for the treatment of advanced cancers due to its overexpression in cancer compared to normal tissue. However, the current therapies to target survivin have unclear mechanisms of action most likely due to pleiotropic effects on other apoptosis-inducing pathways. Furthermore, the most studied inhibitor of survivin, YM155, is associated with serious adverse events without demonstrating increases in efficacy [22,23]. YM155 as a monotherapy is most likely not an option for clinical applications in treating synovial sarcoma. There is potential in treating synovial sarcoma and other survivin-expressing cancers with YM155 in combination with another targeted therapy. We have examined YM155 in combination with BCL2 inhibitors and found them to be minimally synergistic. Other studies are needed to find effective combinations. Additionally, more inhibitors of survivin are needed to dissociate the negative adverse events from the therapeutic benefits of targeting this protein in cancer.

In this study, we have demonstrated the potential indication utilizing YM155 in synovial sarcomas by applying YM155 to both in vitro and in vivo models of synovial sarcoma. The decrease in *Birc5* and survivin expression, the induction of apoptosis, and the decrease in cell viability were clearly detected in synovial sarcoma upon YM155 exposure. However, less clear was the simultaneous enrichment of histone tail modifications at the *BIRC5* promoter that traditionally are thought to promote repression (H3K27me3) or induce gene expression (H3K27ac).

This is not the first study to demonstrate concurrent increases in H3K27me3 and H3K27ac. In a study of hepatocarcinoma, other researchers observed through protein staining that both histone modification markers increased in a subset of samples and that elevated levels of these markers were associated with a poorer prognosis [29]. Increases of H3K27me3 and H3K27ac were not seen in the same nuclei of a cell, but in neighboring cells. Likewise, we are likely sampling two mixed populations of cells within our synovial sarcomas. While it would be parsimonious to assign the H3K27ac increase to non-cancerous cells in the tumor microenvironment, this study indicates that it is not solely non-cancerous that are responsible for the H3K27ac enrichment. However, we have previously measured the percentage of the population of cancerous cells and found it to be between 70%–95% in the non-metastatic models [20,30]. Further, normal tissue’s contribution to the H3K27ac increase does not explain the H3K27ac increase observed in the in vitro experiments using HS-SY-II established cell lines.

Survivin has demonstrated biphasic expression upon constant exposure to apoptosis-inducing drugs in hepatocytes [28]. This biphasic expression is thought to be a result of the interconnections of survivin and cell cycle proteins that are specifically expressed during G2/M phase [31]. What we could be observing are cells in different phases of the cell cycle, which add additional regulation of the *Birc5* gene. Despite the heterosynchronous state of the histone modifications, it is clear that apoptosis prevails. In response to decreased survivin expression, synovial sarcoma cells initiate feedback loops that increase expression of NFκB.

NFκB is a transcription factor that is ubiquitously expressed throughout the body and is upregulated in cancer in response to receptor tyrosine kinases and other autocrine and paracrine signaling events [32]. Others have demonstrated that inhibition of NFκB using the compound BAY 11-7082 leads to a decrease in survivin levels [28]. We have demonstrated that NFκB is significantly upregulated in response to YM155 and that it loosely associates with the *Birc5* promoter. The association of NFκB with the *Birc5* promoter did not change in the presence of YM155. While YM155 could be interacting with NFκB to regulate survivin expression, another study demonstrated indirect effects of YM155 on ILF3/NF110 induced *Birc5* expression [33]. The direct target of YM155 remains unproven, but the effects on epigenetic changes at the *Birc5* promoter are evident in synovial sarcoma.

Knowing that epigenetic regulation of the *Birc5* promoter is essential to YM155 activity, it would be interesting to test the combination of histone acetyltransferase (p300/CBP) inhibitors or demethylase (JMJD3) inhibitors with YM155 in the treatment of cancers listed in Table 1. The synergy of these approaches could overcome the concomitant expression of H3K27ac and H3K27me3 to predominantly favor repression of the *Birc5* promoter, resulting in a more sensitive induction of apoptosis in cancer.

## 5. Conclusions

Synovial sarcoma is another survivin-expressing cancer and as such survivin can be a viable molecular target to treat patients with synovial sarcoma. However, the most widely used survivin inhibitor, YM155, exhibits adverse effects in patients and indirectly affects survivin/*Birc5* levels through an ambiguous mechanism of action. We have demonstrated that histone modifications are altered in synovial sarcoma cells at the *Birc5* promoter upon YM155 administration. Because YM155 exerts its effects through epigenetic regulation of the *Birc5* promoter, administering YM155 in combination with histone acetyltransferase and DNA demethylase inhibitors might prove efficacious. However, without a precise mechanism of selectively targeting the *Birc5* promoter with these epigenetic modifiers, a more productive approach may be to screen for novel survivin inhibitors with a more direct mechanism of action. 

## Figures and Tables

**Table 1:** Summary of clinical trials using YM155 in cancer.

Study Identifier	Number of Patients	Phase	Disease	Date Ended
NCT01023386	6	phase 1	cancer	2010
NCT00818480	10	phase 2	prostate cancer, melanoma, non-Hodgkin′s Lymphoma	2012
NCT01007292	43	phase 2	non-Hodgkin′s Lymphoma	2015
NCT01009775	64	phase 2	melanoma	2012
NCT01038804	101	phase 2	breast cancer	2013
NCT00498914	41	phase 2	lymphoma	2009
NCT01100931	42	phase 1/2	NSCLC ^2^, solid tumors	2015
NCT00514267	32	phase 1/2	prostate cancer, tumors	2015
NCT00281541	29	phase 2	melanoma	2012
NCT00328588	37	phase 2	NSCLC ^2^	2008
NCT00257478	NA ^1^	phase 2	prostate cancer	2007

^1^ No data available. ^2^ Non-small Cell Lung Cancer.
